# A dosimetry innovation using hybrid pixel detectors in interventional theatres

**DOI:** 10.1038/s41598-026-49696-5

**Published:** 2026-04-22

**Authors:** T. Genetay, T. Otto, F. Bochud, P. Carbonez, J. Damet

**Affiliations:** 1 CERN, European Organization for Nuclear Research, Geneva, 1211 Switzerland; 2 Institute of Radiation Physics, Lausanne University Hospital and Lausanne University, Lausanne, Switzerland; 3 Department of Radiology, University of Otago, Christchurch, New Zealand

**Keywords:** Engineering, Health care, Medical research, Optics and photonics, Physics

## Abstract

Healthcare professionals using fluoroscopic imaging systems rank among the most highly exposed workers to ionising radiations, yet current radiation protection detectors fail to provide accurate measurements in these scenarios. The pulsed structure of the X-ray beam produced by these medical devices poses significant challenges to conventional instruments in measuring and characterising time-structured radiation fields. Furthermore, radiation protection detectors consistently overestimate staff exposure at these low energies (< 100 keV) due to the inherent limitations of the dosimetric system currently in-use. In this study, we present a new hybrid pixel detector, originally developed for particle tracking and timing measurements in high-energy physics experiments at CERN, to overcome the above-mentioned challenges. By integrating single-photon energy measurement and precise timing capabilities, the detector enables highly accurate dose quantification across a broad range of diagnostic X-ray conditions while simultaneously resolving the spectral structure of radiation fields. Our findings demonstrate the detector’s suitability for advanced dosimetry assessment in clinical environments. It uniquely measures photon fluence and energy spectra in clinical X-ray fields, enabling accurate, pulse-independent determination of any exposure-related quantity. By giving access to fundamental radiation field parameters, this work lays the foundation for next-generation detectors that enhance staff safety and advance radiation epidemiology.

**Supplementary Information:**

The online version contains supplementary material available at 10.1038/s41598-026-49696-5.

## Introduction

Over the last years, the use of X-rays has expanded across a wide range of medical specialities far beyond radiology departments, becoming a major concern in medical radiation protection, particularly during interventional procedures^1^. Both the number and the complexity of these procedures continue to rise, leading to more frequent use of X-ray imaging in clinical practice^1–3^. While X-ray guided interventions benefit patients by reducing tissue injuries and shortening hospital stays^4,5^, the medical staff are routinely exposed to scattered radiation generated when the primary X-ray beam interacts with patient tissues. As a result, medical personnel performing X-ray guided interventions are among the most occupationally exposed workers worldwide^6^.

Chronic exposure to ionising radiation, even at low levels, is associated with increased health risk. Such exposure may indeed cause health effects, including radiation-induced cancer and cardiovascular diseases^7,8^. Epidemiological and occupational surveys have also demonstrated a higher prevalence of lens opacities and early-stage cataracts in medical staff exposed to scattered radiation from fluoroscopic systems^9–11^. Continuous, accurate monitoring of occupational exposure is therefore essential to support effective radiation protection measures, such as shielding or time restrictions.

The temporal structure of the pulsed primary beam produced by the fluoroscopic devices, with pulse length ranging from 0.1 to 10 ms, is preserved in the scattered radiation field. This characteristic makes accurate dosimetry challenging for active, direct reading detectors. Moreover, at the diagnostic X-ray energies (20–100 keV), conventional radiation protection detectors overestimate staff exposure by approximately a factor of two^12–15^. These two limitations can be addressed with a novel spectral detector, the Timepix4 hybrid pixel detector (HPD). Its pixelated structure provides a time resolution of 195 ps per pixel^16^, enabling accurate measurements in pulsed X-ray fields. Its spectral capabilities enable to assess a new operational quantity which closely estimate the dose actually delivered to the professional, while providing energy-resolved information that is currently not accessible with conventional radiation protection detectors.

### Dose quantities in radiological protection

The evaluation of the exposure level of the workers and the associated health risk requires the use of appropriate quantities. To this purpose, the International Commission on Radiological Protection (ICRP) introduced the concept of effective dose, *E*, expressed in sievert, which can be used as a proxy of the overall risk of cancer induction associated with whole-body exposure^17,18^. Effective dose is inherently a computational, risk-based protection quantity, though it is not a measurable physical quantity. To address the need of practical monitoring, the International Commission on Radiation Units and Measurements (ICRU) introduced operational quantities in their reports 39 and 51^19,20^, which serve as measurable surrogates for effective dose. These quantities however overestimate the effective dose by approximately a factor of two for photon energies below 100 keV^14,15,19–21^, which is the range of interest in X-ray imaging. Indeed, the current definition of operational quantities assumes that all photons interact at a depth of 10 mm, which enhances the contribution of low-energy photons.

To address these limitations, the ICRU and the ICRP jointly proposed a new set of operational quantities designed to provide a more accurate approximation of effective dose across a broad range of energies in ICRU Report 95^21^. These quantities are based on the same realistic anthropomorphic phantoms underlying effective dose^22^, offering improved dosimetric accuracy while preserving the necessary conservatism for radiation protection. Their adoption would allow a more precise and realistic assessment of radiation-related health risks to medical staff. The transition to these new standards has sparked substantial debate within the community and represents a significant challenge.

### Limitations of radiation protection detectors

Existing radiation protection detectors measure current operational quantities but cannot measure the new operational quantities such as the ambient dose H*, leading to the necessity of new detection systems and/or calibration methods^12,13,23–25^. Adapting these detectors to the new system is technically challenging. Ideally, a single detector able to accurately measure both systems of operational quantities would ensure a smooth and consistent transition between them. Achieving this dual capability requires direct knowledge of the energy of each photon interacting in the detector, something that conventional radiation protection detectors cannot provide.

Yet it remains the fact that commonly used active detectors struggle with the temporal characteristics of scattered X-ray field encountered in interventional theatres. Pulsed X-ray fields generated by fluoroscopic devices, often lead to dead-time losses and/or saturation effects in current active detectors used in radiation protection^26–30^. These limitations compromise accurate exposure assessment in interventional theatres.

### Timepix4 hybrid pixel detector

In this study, we propose a new type of HPD that overcomes these limitations. Originally developed for particle tracking in high-energy physics^31^, their pixelated structure enables single-photon processing: each pixel is connected to an individual readout channel within an application-specific integrated circuit (ASIC), allowing simultaneous detection, energy measurement, and timestamping of incoming photons. These capabilities directly address key challenges encountered in dosimetry for pulsed and highly heterogeneous low-energy X-ray fields.

HPDs have already contributed to major advances in medical imaging, most notably through the development of photon-counting computed tomography, which combines improved image quality with reduced patient dose^32^. The Timepix family of detectors, developed within the Medipix collaborations hosted at CERN, extends these capabilities to radiation measurement. Timepix detectors integrate energy and time-of-arrival information for individual photons^33^, providing detailed insight into both the spectral and temporal structure of X-ray fields. They have already contributed to greatly improve dosimetry of space crews^34,35^, notably during the Artemis I lunar mission^36^. The present study demonstrates, for the first time, the accurate, real-time determination of operational quantities from the Timepix4 detector spectral measurement. It is therefore the first detector capable of simultaneously providing both spectral and operational quantities measurement under reference clinical conditions relevant to the interventional radiology energy range. These capabilities open new perspectives for accurate exposure assessment in interventional theatres and uniquely enable the determination of both current and newly proposed operational quantities within a single measurement.

## Results

**Fig. 1:**
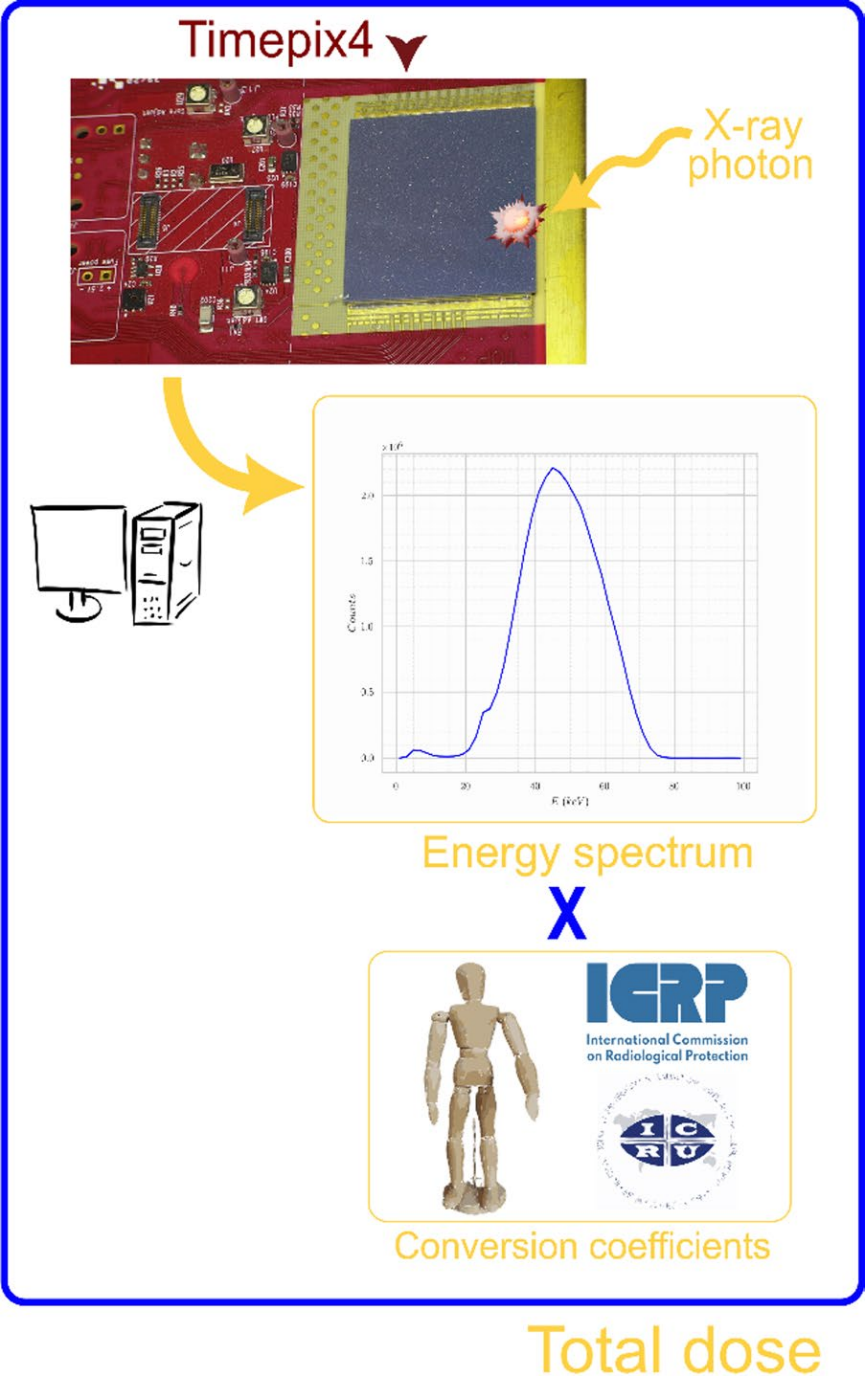
Scheme representing the process to compute total dose from X-ray photons measured with the Timepix4 detector.

For each acquisition, the detector provides data that enables real-time computation of the energy spectrum as well as reconstruction of the temporal structure of the incident X-ray field. These spectral measurements were used to derive the ambient dose equivalent H*(10) and the ambient dose H*, which estimate the effective dose *E* in the current and newly proposed radiation protection dosimetry systems, respectively, in addition to other quantities defined in the Methods section. The full workflow is summarised in Fig. 1: X-ray photons are recorded by the Timepix4 detector, reconstructed into an energy spectrum using the analysis workflow described in the Methods section, and the resulting photon fluence spectrum is converted into the corresponding dose quantities by applying the appropriate conversion coefficients. The performance of the detector was assessed in beam qualities representative of those encountered in interventional theatres, as defined in the standard IEC 61267 (RQR and RQA)^37^. All measurements were compared with reference values obtained using an ionisation chamber for each beam quality. Although the newly proposed operational quantities cannot yet be measured under operational conditions, their values under reference conditions can be determined by applying a single conversion factor to a physical quantity measured for a given beam quality, when using a reference instrument such as the ionisation chamber mentioned above and described in the Methods section. It was therefore possible to precisely assess the performance of the Timepix4 detector for the measurement of the operational quantities.

**Fig. 2:**
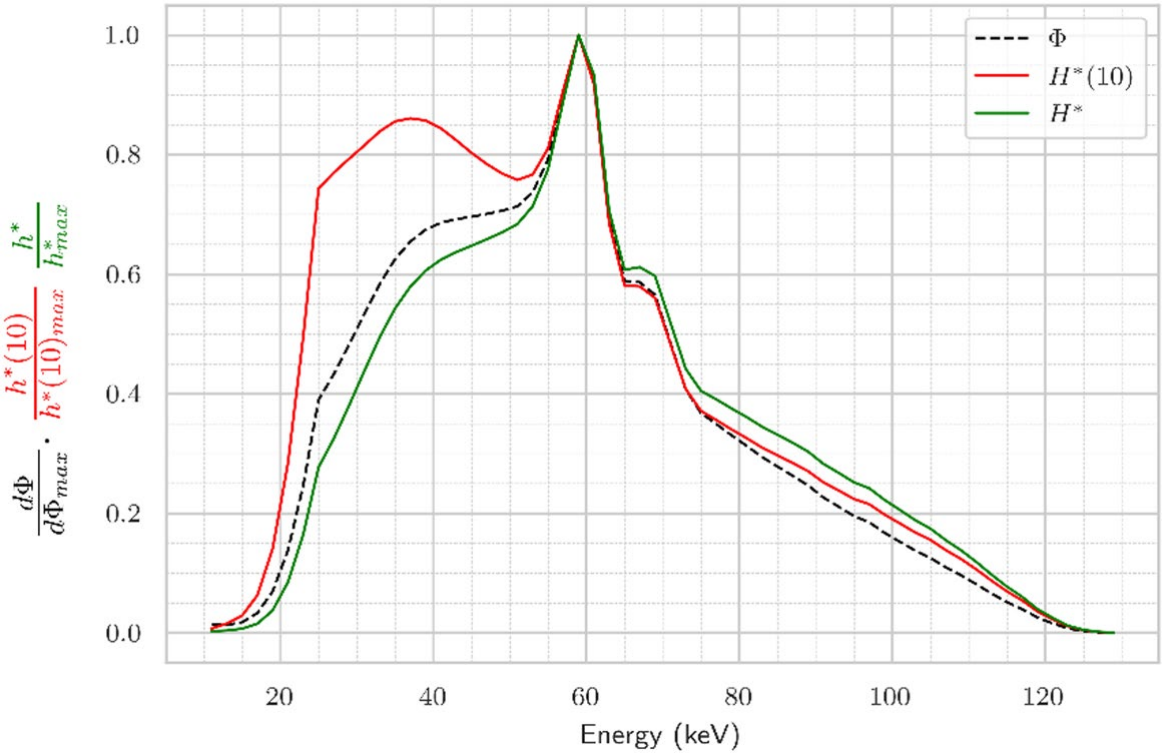
Fluence spectrum as a function of energy (dotted black), distribution of H*(10) (red), and distribution of H* (green) as a function of photon energy for the RQR9 beam quality. All distributions are normalised to their respective maxima.

Figure 2 shows the spectra expressed in terms of photon fluence *Φ*, as well as the current operational quantity H*(10), and the new one H* obtained for the RQR9 beam quality at a tube current of 1 mA. All distributions are normalised to their respective maxima for visual clarity. Similar spectra were obtained for all measurements performed with the Timepix4 detector, as well as for the other quantities described in the Methods section. Only photon energies above 10 keV were included in the computation of dosimetric quantities to minimise the influence of electronic noise and threshold-related effects at low energies.

**Fig. 3:**
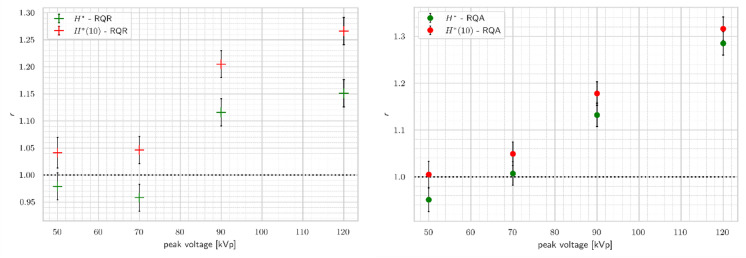
Absolute response of the Timepix4 detector for the measurement of H*(10) (red) and H* (green) as a function of the tube’s peak kilovoltage for the RQR (crosses, left panel) and the RQA (dots, right panel) spectral series.

Figure 3 shows the absolute response *r* of the Timepix4 detector for H*(10) and H* as a function of the X-ray tube kilovoltage peak (*kVp)*, computed as the ratio between the reference value measured by an ionisation chamber and the value extracted from Timepix4 spectra. The *kVp* corresponds to the maximum voltage applied to the X-ray tube and therefore defines the upper limit of photon energies in the emitted spectrum.

## Discussion

The Timepix4 detector demonstrates accuracy within 5% for both H*(10) and H* up to 70 kVp, corresponding to a maximum photon energy of 70 keV. At higher energies, the detector’s absolute response gradually decreases, reaching deviations of approximately 20% for both quantities at 120 kVp. Overall, the Timepix4 showed a strong stability throughout the measurements, with a standard deviation not higher than 0.5% for a single beam quality.

These deviations at higher energies arise from spectral artifacts, inherent to the detector. As shown in a previous study^38^, most discrepancies in spectra originate from X-ray fluorescence produced by bonding materials and the low intrinsic detection efficiency of the 300 μm silicon sensor. The impact of the fluorescence can be clearly observed in Fig. 2, where a discontinuity appears around 25 keV, corresponding to the tin used in the bump bonds of the Timepix4 detector. This effect leads to a localised overestimation of quantities dominated by contributions from lower energy photons, explaining the slight overestimation of H*(10) compared to H*. The overall performance of Timepix4 could be further improved by refining the reconstruction algorithm to enhance results in the high-energy region and suppress the fluorescence-induced peak, highlighting the significant potential of this detector for area monitoring during interventional procedures. Improved Monte Carlo modelling of the detector would help suppress non-linearities in the low-energy region of the spectrum. Improved reconstruction methods, such as Bayesian deconvolution^39^, may also enhance the reconstruction of the higher-energy region, but would critically need a more accurate detector model. It should be noted that the proposed approach is not suitable for higher-energy photons, as the silicon sensor’s detection efficiency is insufficient for photons above 150 keV. An alternative detection medium, such as cadmium telluride (CdTe), could potentially extend the measurable range up to 500 keV, but would presents significant technical challenges. In particular, the increased detection efficiency of CdTe would come at the cost of a count rate far exceeding the current system bandwidth by more than one order of magnitude, making such measurements impractical without substantial hardware upgrades. Furthermore, a CdTe sensor would also introduce additional K-fluorescence photons within the sensor itself, in the 20–30 keV energy range, making the improvement of the reconstruction algorithm even more critical.

To the authors’ knowledge, the Timepix4 detector is currently the only active detector capable of directly measuring the operational quantities defined in ICRU Report 95 with this level of accuracy in the low-energy range. All values presented in Fig. 3, as well as those reported for the quantities in the Supplementary Materials, fall within the relative response limits specified by IEC 60846-1^40^. The Timepix4 detector therefore represents a unique bridge between the current and the newly proposed radiation protection systems and is already compliant with existing standards.

The Timepix4 detector provides accurate measurements of photon fluence and X-ray energy distribution in clinically relevant X-ray fields^38^. This unique ability enables not only detailed field characterisation and accurate determination of operational quantities relevant to whole-body exposure, but also the calculation of any dose quantity for which conversion coefficients are available. As such, the Timepix4 detector has the potential to serve as a universal, long-term solution adaptable to any dosimetric quantity system. While most conventional detectors exhibit poorer performances under pulsed X-ray fields^41^, the Timepix4 detector is inherently insensitive to the temporal structure of radiation fields encountered during interventional procedures^42^. These combined capabilities place it well ahead of existing radiation protection detectors for measurement in such theatres.

Moreover, its relative response over the tested range is already comparable to or better than that of existing systems^28,41,43–46^, even exceeding their performance for tube voltages up to 70 kVp, while maintaining equivalent accuracy with the newly proposed operational quantities.

### Perspectives

This study presents a unique detector system capable of measuring clinically relevant energy spectra from which operational quantities can be directly and accurately derived, for low-energy X-ray photons (10 to 120 keV). Fluence-based dosimetry potentially enables the determination of any dose quantity, regardless of present or future dosimetric framework, provided that appropriate fluence-to-dose conversion coefficients are available and the measured fluence is accurate. As such, it offers both flexibility and long-term robustness for an accurate protection of medical personnel during interventional procedures. The strong performances of the Timepix4 detector for particle tracking could be investigated in the future for use in beta dosimetry, which could potentially greatly improve dosimetry in mixed radiation fields.

Beyond its implications in X-ray metrology and dosimetry, the Timepix4 detector could have a significant impact on radiation epidemiology, which aims to quantify the relationship between organ dose and the occurrence of radiation-associated diseases^47^. Indeed, understanding the physical characteristics of occupational radiation fields is fundamental to establishing robust exposure-effect relationships. Detailed spectral and temporal data enable reassessment of historical exposures and improve epidemiological risk estimates. This enhanced dosimetric precision is critical for resolving uncertainties in low-dose radiation health effects, particularly for cardiovascular disease and lens opacities. Current epidemiological studies rely on operational quantities measured by personal dosimeters, which are weighted according to a specific dosimetric system. They must be converted to organ dose, using additional hypotheses about the energy spectrum of radiation. By providing direct access to the energy spectrum and fluence, fundamental physical parameters of the radiation field, the Timepix4 detector offers a new source of reliable input data for future epidemiological studies. This capability has the potential to improve our understanding of the health effects of chronic exposure to pulsed X-ray fields and to support studies based on quantities that will remain unchanged over time.

Overall, the Timepix4 detector represents a unique detector to achieve a more accurate and reliable dosimetry. By opening access to physical quantities intrinsically linked to the radiation field, it promises to improve the protection of medical staff in interventional theatres while contributing to more robust scientific knowledge on chronic exposure to low-dose X-ray pulsed fields.

### Methods

#### The Timepix4 detector

The Timepix4 detector is a Hybrid Pixel Detector (HPD) developed within the framework of the Medipix4 collaboration, hosted at CERN. It represents the latest generation of the Timepix family^16^. The detector consists of a matrix of 448× 512 square pixels, each with a 55 μm pitch, and it can process up to 80 million hits per second. Each pixel is connected to its own readout channel within the Application-Specific Integrated Circuit (ASIC), allowing for single-photon processing. In this study, the Timepix4 was bump-bonded to a 300 μm thick silicon sensor, which was biased at 100 V using a Keithley 2400 source-meter (Tektronix, Beaverton, USA).

In this study, the detector was operated in data-driven mode that collects a raw data set whenever an interaction occurs. Under this configuration, data from any single pixel is collected exclusively when its detected signal surpasses a predefined threshold. The detection threshold was set to 3.6 keV per pixel, representing a compromise between minimizing the number of noisy pixels requiring masking and reducing energy losses due to charge sharing, as further described in Subsection 1.2.

Each recorded data set contains the pixel coordinates, the time of arrival of the particle, and the time over threshold (*ToT*), which is proportional to the energy deposited in the pixel. While the *ToT* parameter is proportional to the deposited energy, an individual energy calibration must be performed for each pixel within the matrix. The *ToT*-to-energy calibration procedure was performed using a well-established method for Timepix4 detectors, based on internal voltage test-pulses^48–50^. Tests-pulses consist of electronic signals directly injected into each pixel, simulating the interaction of incoming photons. The calibration procedure was performed using pulse amplitudes corresponding to energy deposits between 3.6 keV and 43.2 keV, distributed over 20 discrete values, covering the energy range relevant to diagnostic radiology. Consistent with previous studies, the Timepix4 detector shows linear response above 10 keV^51^, confirmed with accurate comparisons with reference RQR spectra up to 120 keV^38^.

In this study, the Timepix4 detector was operated in combination with the SPIDR4 readout system (Speedy Pixel Readout 4, Nikhef, Amsterdam, The Netherlands) and the Datapix4 acquisition software (INFN Ferrara, Ferrara, Italy)^52^ for data collection.

### Data analysis

In the present work, Timepix4 was employed as a spectrometer-like detector. To reconstruct the energy spectra from the raw Timepix4 data, a complete data processing workflow, described in detail by Genetay et al^38^, was used. This pipeline, developed in Python, integrates algorithms for charge-sharing event reconstruction and intrinsic detection efficiency calibration, enabling accurate spectral reconstruction from the detector’s raw output.

When an X-ray photon interacts with the detector, the resulting charge cloud can extend beyond a single pixel, causing the energy from one photon to be deposited across several neighbouring pixels^53,54^. The charge sharing effect must be carefully considered during data analysis to ensure accurate photon energy measurements. The charge sharing reconstruction algorithm is based on a density-based clustering approach, implemented using the scikit-learn Python package^55^, and was successfully benchmarked against Monte-Carlo simulations for the RQR serie in Genetay et al^38^.

The intrinsic detection efficiency, hereafter referred to as detection efficiency, represents the sensor’s ability to attenuate and absorb incoming X-ray photons. Due to the material properties of silicon, complete absorption cannot be achieved across the full interventional radiology energy range, making a precise characterisation of detection efficiency essential for accurate fluence and dose measurements. The detection efficiency was determined using Geant4 Monte-Carlo simulations^56^, in which a 300 μm thick silicon layer was exposed to monoenergetic photons from 5 to 150 keV in 2 keV steps. The present model therefore integrates solely the sensor rather the complete detector model, including the ASIC and the bump-bonds. These simulations yield the probability of an incident photon depositing energy within the sensor, ranging from 0 keV up to the full photon energy. Each energy bin is therefore corrected for the total absorption probability, while recursively removing the Compton scattering continuum associated with each energy bin. An extensive description of this unfolding algorithm, as well as its performances, can be found in Genetay et al^38^.

For each measurement, the energy spectrum is obtained. This spectrum can be expressed as a discrete sum of photon fluence *Φ(E)* over fixed energy bins, with *Φ* the total particle fluence in the spectrum, as described by Eq. (1):

1 $$\:{\Phi\:}\:=\:\sum\:_{E}{\Phi\:}\left(E\right)$$

While conventional photon dosimetry is primarily based on integrating the energy deposited in the detector medium, later converted to absorbed dose in water *D* or to operational quantities, the present work proposes to take advantage of the unique capacity of Timepix detectors to distinguish individual photon events.

This approach enables direct computation of both the air kerma ($\:{K}_{a}$) and operational quantities from the measured photon fluence, as described in ICRU Report 95^21^. Equation 2, derived from Eq. 1, illustrates the calculation of $\:{K}_{a}$, which can similarly be applied to determine operational quantities. Here, $\:{k}_{{\Phi\:},\mathrm{E}\:}$represents the monoenergetic conversion coefficients between photon fluence and $\:{K}_{a}$, provided in various reports such has ICRU Report 95 or ICRP Publication 74:

2 $$\:{K}_{a}=\:\sum\:_{E}{\Phi\:}\left(E\right){k}_{{\Phi\:},E}\left(E\right)$$

From a single Timepix4 spectral measurement, a total of seven dosimetric quantities is computed. These quantities, along with the corresponding bibliographic sources for the conversion coefficients, are listed in Table 1. Following the recommendation of Behrens and Otto^57^, a standard cubic spline interpolation was applied to interpolate the dose conversion coefficients at the central energy of each bin. To allow comparison with commonly used detector systems, the quantities are further normalised by the active area of the detector. Moreover, a 10 keV threshold is applied on the energy spectrum to mitigate the impact of low-energy threshold noise and fluorescence lines. To validate the accuracy of the proposed method, the analysis scripts were applied to the reference energy spectra published by Ankerhold^58^, in terms of $\:{K}_{a}$ and $\:{H}^{*}\left(10\right)$. A detailed residual analysis between the published and computed distributions is provided in the supplementary materials.

**Table 1:** Operational dosimetry quantities measured with the Timepix4 detector and their respective sources for the conversion coefficients between photon fluence $\:{\boldsymbol{\Phi\:}}_{\boldsymbol{E}}$ and these dosimetry quantities as well as for the RQR and RQA beam qualities from Int. Standard IEC 61,267.

Dosimetry quantity	Conversion coefficients for monoenergetic photons	Conversion coefficients for X-ray spectra of the RQR/RQA series^37^
Total air kerma $\:{\boldsymbol{K}}_{\boldsymbol{a}}$ [Gy]	ICRU report 95^21^	-
Ambient dose $\:{\boldsymbol{H}}^{\boldsymbol{*}}$[Sv]	ICRU report 95^21^	RQR: Behrens and Otto^57^ RQA: Yadav et al^59^.
Ambient dose equivalent $\:{\boldsymbol{H}}^{\boldsymbol{*}}\left(10\right)$ [Sv]	ICRP Publication 74^60^	U. Ankerhold^58^
Directional absorbed dose in local skin $\:{\boldsymbol{D}}_{\boldsymbol{l}\boldsymbol{o}\boldsymbol{c}\boldsymbol{a}\boldsymbol{l}\:\boldsymbol{s}\boldsymbol{k}\boldsymbol{i}\boldsymbol{n}}^{\boldsymbol{{\prime\:}}}$ [Gy]	ICRU report 95^21^	RQR: Behrens and Otto^57^ RQA: Yadav et al^59^.
Directional dose equivalent at 0.07 mm depth $\:\boldsymbol{H}\boldsymbol{{\prime\:}}(0.07)$	ICRP Publication 74^60^	U. Ankerhold^58^
Directional absorbed dose in the lens of the eye $\:{\boldsymbol{D}}_{\boldsymbol{l}\boldsymbol{e}\boldsymbol{n}\boldsymbol{s}}^{\boldsymbol{{\prime\:}}}$ [Gy]	ICRU Report 95^21^	RQR: Behrens and Otto^57^ RQA: Yadav et al^59^.
Directional dose equivalent at 3 mm depth $\:\boldsymbol{H}\boldsymbol{{\prime\:}}\left(3\right)$ [Sv]	Computed with conversion coefficients per unit of total air kerma from Šolc & al^61^. and total air kerma per unit of photon fluence from ICRU report 95^21^.	Yadav et al^59^.

### Measurements at the calibration laboratory

The accuracy of Timepix4 in estimating the quantities described in Table 1 was assessed using reference radiation qualities. This approach ensures that the X-ray field is well characterised and that the measurements obtained with Timepix4 can be directly compared with those from a reference ionisation chamber. To closely replicate the X-ray spectra encountered around fluoroscopy systems, the field should ideally originate from scattering, be pulsed, and exhibit a comparable energy distribution. Although pulsed X-ray fields are defined in ISO/TS 18090^62^ and IEC 63050^63^ standards, their availability remains limited. Therefore, the RQR and RQA beam qualities defined in IEC 61267^37^ were selected, as they provide a broad energy distribution representative of spectra typically observed in interventional rooms, while acknowledging that their spatial and temporal characteristics differ from those of actual scattered fields.

The measurements were performed at the accredited dosimetry laboratory of the University Hospital of Lausanne. The characteristics of the beam qualities available at the laboratory are listed in Table 2. These beam qualities were selected among the RQR and RQA series to evaluate the detector performance across the full energy range typically encountered around fluoroscopy systems, and to cover a variety of spectral shapes. The X-ray tube TU 320-D03 (Yxlon International GmbH, Hamburg, Germany) installed at the laboratory was used for all irradiations. For each beam quality, measurements were performed at a beam current value ensuring between 10 and 15 millions of photons measured by the detector, with an acquisition time of 30 s, to achieve sufficient reliability on the results while maintaining consistent comparisons between the different beam qualities. This number of photons corresponds to the following beam current values:

- RQR3 and RQR5: 1 mA.
- RQR7 and RQR9: 0.5 mA.
- RQA beam qualities: 10 mA.

For each combination of beam quality and tube current, measurements were performed at a source-to-detector distance of 2.5 m. This configuration was chosen to avoid readout saturation while maintaining sufficient beam homogeneity and adequate counting statistic. Each measurement was repeated three times to verify the stability of the system.

**Table 2:** RQR and RQA beam quality definitions in Int. Standard IEC 61,267 and the additional aluminium filtration to achieve these characteristics determined at the calibration laboratory of the University Hospital of Lausanne.

Beam code	Voltage U (kV)	1 st HVL (mm Al) (IEC 61267)	Add. Filtration (mm Al)
RQR3	50	1.78	2.62
RQR5	70	2.58	2.62
RQR7	90	3.48	3.60
RQR9	120	5.00	3.60
RQA3	50	3.8	12.62
RQA5	70	6.8	23.62
RQA7	90	9.2	33.60
RQA9	120	11.6	43.60

To validate the estimation of the quantities listed in Table 1 obtained with the Timepix4 detector, results were compared against reference measurements performed with an ionisation chamber. These measurements were performed in $\:{K}_{a}$ using a Radcal 10X5-60E ionisation chamber (Radcal Corp., Monrovia, USA) connected to a Radcal 9015 electrometer, both traceable to the NPL (National Physical Laboratory, Teddington, UK). The laboratory is therefore a secondary standard for $\:{K}_{a}$ measurements, and each chamber measurement was corrected using the appropriate beam quality factors while accounting for temperature and pressure effects. For each beam quality and tube current, three measurements were performed at a source-to-detector distance of 2.5 m and an acquisition time of 30 s. For the RQA beam qualities, the acquisition time was increased to 90 s to ensure adequate measurement stability. Ionisation chamber measurements were then converted to the operational quantities listed in Table 1 using the conversion coefficients provided in Table 3. These conversion coefficients are defined for a specific beam quality *R* per unit of $\:{K}_{a}$, and correspond to the following quantities:

- $\:{\boldsymbol{h}}_{\boldsymbol{K}}^{\boldsymbol{*}}\left(\boldsymbol{R}\right)$ to determine the ambient dose $\:{H}^{*}$.
- $\:{\boldsymbol{d}}_{\boldsymbol{l}\boldsymbol{e}\boldsymbol{n}\boldsymbol{s}~\boldsymbol{K}}\left(\boldsymbol{R}\right)$ to determine the directional absorbed dose to the lens of the eye $\:{{D}^{{\prime\:}}}_{lens}$.
- $\:{\boldsymbol{d}}_{\boldsymbol{l}\boldsymbol{o}\boldsymbol{c}\boldsymbol{a}\boldsymbol{l}~\:\boldsymbol{s}\boldsymbol{k}\boldsymbol{i}\boldsymbol{n}~\:\boldsymbol{K}}\left(\boldsymbol{R}\right)\:$to determine the directional absorbed to the local skin $\:{D}_{local~\:skin}^{{\prime\:}}$.
- $\:{\boldsymbol{h}}^{\boldsymbol{*}}\left(10,\boldsymbol{R}\right)$ to determine the ambient dose equivalent $\:{H}^{*\:}\left(10\right)$.
- $\:\boldsymbol{h}\boldsymbol{{\prime\:}}(3,\boldsymbol{R})$ to determine the directional dose equivalent at 3 mm $\:H{\prime\:}\left(3\right)$.
- $\:{\boldsymbol{h}}^{\boldsymbol{{\prime\:}}}\left(0.07,\boldsymbol{R}\right)$ to determine the directional dose equivalent at 0.07 mm $\:H{\prime\:}\left(0.07\right)$.

**Table 3:** Spectrum averaged conversion coefficients between dosimetry quantities and $\:{\boldsymbol{K}}_{\boldsymbol{a}}$ for RQR/RQA beam qualities.

Beam code	$\:{\boldsymbol{h}}_{\boldsymbol{K}}^{\boldsymbol{*}}\left(\boldsymbol{R}\right)$ [Sv/Gy]	$\:{\boldsymbol{d}}_{\boldsymbol{l}\boldsymbol{e}\boldsymbol{n}\boldsymbol{s}~ \boldsymbol{K}}\left(\boldsymbol{R}\right)$ [Gy/Gy]	$\:{\boldsymbol{d}}_{\boldsymbol{l}\boldsymbol{o}\boldsymbol{c}\boldsymbol{a}\boldsymbol{l}\:~\boldsymbol{s}\boldsymbol{k}\boldsymbol{i}\boldsymbol{n}~\boldsymbol{K}}\left(\boldsymbol{R}\right)\:$ [Gy/Gy]	$\:{\boldsymbol{h}}^{\boldsymbol{*}}\left(10,\boldsymbol{R}\right)$ [Sv/Gy]	$\:\boldsymbol{h}\boldsymbol{{\prime\:}}(3,\boldsymbol{R})$ [Sv/Gy]	$\:\boldsymbol{h}\boldsymbol{{\prime\:}}(0.07,\boldsymbol{R})$ [Sv/Gy]
RQR3	0.4208	1.0981	1.2769	1.01	1.093	1.20
RQR5	0.6121	1.2191	1.3869	1.16	1.260	1.26
RQR7	0.7788	1.3065	1.4747	1.26	1.329	1.31
RQR9	0.9571	1.3873	1.5578	1.36	1.395	1.36
RQA3	0.7210	1.2880	1.4400	1.37	1.388	1.34
RQA5	1.1180	1.4750	1.6540	1.63	1.578	1.48
RQA7	1.3040	1.5390	1.7430	1.70	1.636	1.52
RQA9	1.3680	1.5390	1.7340	1.70	1.626	1.53

From these measurements, the relative response *r* of the Timepix4 detector to $\:{K}_{a}$ and the operational quantities was evaluated using Eq. 3, where $\:{M}_{TPX4}$ denotes the value measured by Timepix4 and $\:{M}_{ref}$ represents the corresponding measurement from the ionisation chamber^40^:

3 $$\:r=\frac{{M}_{TPX4}}{{M}_{ref}}$$

The absolute response *r* was therefore expressed as a function of X-ray tube kilovoltage peak (*kVp)*, in accordance with the recommendations of the International Atomic Energy Agency Technical Report Series No. 457^64^.

The reader should note that a source-to-detector distance of 2.5 m is not considered a reference distance according to the IEC 61267 standard^37^. Although the X-ray beam is generally homogeneous enough to ensure consistent measurements at this distance, small spectral variations may occur, leading to slight deviations in the conversion coefficients. In the ISO 4037:3 standard^65^, the X-ray beam qualities are defined at both 1 and 2.5 m. A comparison of the spectral distributions at 1 m and 2.5 m for the RQA beam qualities is provided in the Supplementary Materials. Due to bandwidth limitations of the Timepix4 detector readout, measurements could not be performed at 1 m for the RQR beam qualities. However, since the RQR and RQA beams were generated using the same tube and exhibit comparable tube voltages, similar spectral behaviour is expected between the two sets of beam qualities, and the comparison with the RQA series is considered sufficient.

### Uncertainties

The assessment of measurement uncertainties is a critical step in dosimetry, as it determines the reliability and validity of the results. To ensure a robust evaluation of the measured quantities, detailed uncertainty calculations were added to the data analysis workflow described in Sect. 1.2. Equation 2 can therefore be reformulated as Eq. 4 where *Φ* represents the photon fluence, *E* is the photon energy, and $\:\left(\frac{{\mu\:}_{en}\left(E\right)}{\rho\:}\right)$ is the energy absorption coefficient per energy bin:

4 $$\:{K}_{a}=\:\sum\:_{E}E\varPhi\:\left(E\right)\left(\frac{{\mu\:}_{en}\left(E\right)}{\rho\:}\right)$$

With the Timepix4 detector, *Φ* and *E* are expressed according to Eqs. 6 and 7, and all contributing terms are summarised in Table 4. These contributions represent all sources of uncertainty affecting the measured quantities obtained with Timepix4. The correction factors introduced in Eqs. 5 and 6, denoted by $\:f$, are set to unity either because their influence on the measurements was found to be negligible or due to insufficient information to assign a more accurate value. They are therefore integrated solely to ensure a comprehensive uncertainty evaluation.

5 $$\:E=f\left(ToT\right)\:.\:{\epsilon}_{Si}.\:{f}_{fit}.\:{f}_{temp}$$

6 $$\:{\Phi\:}\:\left(\mathrm{E}\right)=\:\frac{N\left(E\right)}{{\mu\:}_{ph}\left(E,\:\:300\right)}\:.\:{f}_{ang}\:.\:{f}_{analog}.\:{f}_{digital}.{f}_{temp}$$

The effects of temperature and angular dependence was previously investigated^51^. Temperature variations were shown to have a negligible impact on the practical range of interest, while the angular dependence is negligible under reference conditions as the detector is positioned perpendicular to the X-ray beam aperture. According to ISO 4037:1^65^, minor deviations caused by the conical beam geometry are considered negligible at a source-to-detector distance of 2.5 m. No additional correction is required for the calibration fit, whose accuracy has been validated in earlier studies^48–51^, nor for the clustering algorithm, whose performance was assessed using the RQR serie^38^. Although analog and digital pileup could, in principle, require correction factors depending on the fluence rate, current knowledge of these in Timepix4 remains limited. To minimise their influence, all measurements were conducted under conditions where both effects are expected to be minimal.

Analog pileup refers to the probability that a photon interacts within a pixel while the preamplifier output from a previous photon interaction remains above the detector threshold. In the context of this study, the combination of low intrinsic detection efficiency, 64-bits data packet size, and uniform matrix irradiation keeps this probability below 0.01%. In fact, the current bandwidth available implies that the readout system will saturate before reaching fluence rates were the probability of analog pileup become significant. Conversely, digital pileup occurs when an event is not read out in time before a subsequent interaction, resulting in two events being recorded as one. To prevent this, Timepix4 was always operated below its saturation rate. Each data set includes a dedicated pileup bit, indicating whether a digital pileup occurred. The overall digital pileup probability is computed for each measurement and maintained around 0.12%, ensuring minor impact on fluence determination.

A detailed description of the individual uncertainty contributions and the overall uncertainty calculation for Timepix4 measurements is provided in the Supplementary Materials.

**Table 4:** Description of the contributions to the measurement of *E* and *Φ* with the Timepix4 detector.

Variable	Description
*ToT*	Time over threshold: length of the preamplifier output decay pulse measured by Timepix, proportional to the photon energy.
$\:\boldsymbol{f}\left(\boldsymbol{T}\boldsymbol{o}\boldsymbol{T}\right)$	Calibration function between the *ToT* and the absolute number of charges collected by a single pixel, further described in previous works^49–51,66^.
$\:{\boldsymbol{\epsilon}}_{\boldsymbol{S}\boldsymbol{i}}$	Ionisation energy of the silicon, converting collected charges into energy^67^.
$\:{\boldsymbol{f}}_{\boldsymbol{f}\boldsymbol{i}\boldsymbol{t}}$	Accuracy of the calibration fit, determined by a chi squared test integrated in the Datapix4 software^52^.
$\:{\boldsymbol{f}}_{\boldsymbol{t}\boldsymbol{e}\boldsymbol{m}\boldsymbol{p}}$	Influence of the temperature on energy measurements performed with Timepix, evaluated in a previous work^51^.
$\:{\boldsymbol{f}}_{\boldsymbol{c}\boldsymbol{l}\boldsymbol{u}\boldsymbol{s}\boldsymbol{t}\boldsymbol{e}\boldsymbol{r}\boldsymbol{s}}$	Accuracy of the energy reconstruction of the clustering algorithm, described in a previous work^38^.
$\:\boldsymbol{N}\left(\boldsymbol{E}\right)$	Number of measured photons per energy bin.
$\:{\boldsymbol{\mu\:}}_{\boldsymbol{p}\boldsymbol{h}}(\boldsymbol{E},300)$	Probability of total absorption in the 300 μm silicon sensor, to which the photons are normalised. The normalisation process was described and validated in a previous work^38^.
$\:{\boldsymbol{f}}_{\boldsymbol{a}\boldsymbol{n}\boldsymbol{g}}$	Influence of the angular response on the number of counts measured by the detector, evaluated in a previous work^51^.
$\:{\boldsymbol{f}}_{\boldsymbol{a}\boldsymbol{n}\boldsymbol{a}\boldsymbol{l}\boldsymbol{o}\boldsymbol{g}}$	Influence of the analog pileup on the measured number of photons, evaluated in the dedicated Section in the supplementary materials.
$\:{\boldsymbol{f}}_{\boldsymbol{d}\boldsymbol{i}\boldsymbol{g}\boldsymbol{i}\boldsymbol{t}\boldsymbol{a}\boldsymbol{l}}$	Influence of the digital pileup on the measured number of photons, evaluated using the pileup bit from each data set sent by the detector^16^.

## Supplementary Information

Below is the link to the electronic supplementary material.

Supplementary material 1 (DOCX 47.5 kb)

## Data Availability

Data will be made available on reasonable request.
