# Supplementary material for: A dosimetry innovation using hybrid pixel detectors in interventional theatres
Source: Sci Rep. 2026 Apr 22;16:18657. doi: 10.1038/s41598-026-49696-5 (PMC13270040; doi:10.1038/s41598-026-49696-5)
Supplement: Supplementary file 1 — Supplementary material 1 (DOCX 47.5 kb) [file 41598_2026_49696_MOESM1_ESM.docx]

**Supplementary Information**

**Supplementary Methods**

**Accuracy of the dose computation method**

In 2000, Ankerhold ^1^ published reference spectra expressed in terms of particle fluence ($\Phi_{E}$), total air kerma (*K_a_*), and personal dose equivalent (*H_p_*(10)). To evaluate the accuracy of the method proposed in this study for deriving dose quantities from measured spectra, the same computational procedure was applied to the fluence distributions reported by Ankerhold ^1^ to obtain the corresponding air kerma distributions. These reconstructed *K_a_* spectra were then compared with the reference *K_a_* spectra published by Ankerhold ^1^. The RQR beam qualities were selected for this comparison, as they exhibit the broadest energy distribution among those investigated in this paper. The overall agreement between the reconstructed and reference spectra was expressed by computing the root-mean square (RMS) and the higher absolute residual, both expressed in % relative to the peak value of the spectrum (Table 9). These metrics provide complementary information: the RMS describes the overall spectral deviation, while the maximum residual identifies local discrepancies at individual energies.

**Homogeneity of the X-ray beam**

As mentioned in the material and methods Section, a source-to-detector distance of 2.5 m is not considered a reference distance according to the IEC 61267 standard ^2^. Measurements were performed at 1 m (the only existing reference distance according to IEC 61267 standard) and 2.5 m for the RQA beam quality. Due to bandwidth limitations, measurements could not be performed at 1 m for the RQR beam qualities. However, since the RQR and RQA beams were generated using the same tube and exhibit comparable tube voltages, similar spectral behaviour is expected between the two sets of beam qualities and comparison with the RQA series is considered justified. For each beam quality, spectra acquired with Timepix4 were normalised to their respective maxima to enable direct comparison between data measured at 1 m and 2.5 m (Table 10). Spectral homogeneity was assessed by RMS and the higher absolute residual, both expressed in % relative to the peak value of the spectrum.

**Uncertainties**

As detailed in Section 1.4 of the Material and Methods, the calculation of dose quantities can be expressed by Equation 1, using K_a_ as an example:

$K_{a}=\sum_{ToT} f\left( ToT \right).\epsilon_{Si}.f_{fit}.f_{temp}.\frac{N\left( ToT \right)}{\mu_{ph}\left( ToT,300 \right)}. f_{ang}.f_{analog}.f_{digital}.f_{clusters} .k_{\phi,E}(ToT)$ (1)

The sources of uncertainty and their corresponding values are presented in Table 1. The statistical uncertainties *u(ToT)* and *u(N)* were calculated for each energy bin and averaged over the whole matrix using the test pulse data acquired during energy calibration. As described in Section 1.1 of the Material and Methods, test pulses were generated up to 40 keV. Since the statistical uncertainty was observed to decrease with increasing energy, it was assumed to remain constant for energies above 40 keV. Under reference conditions, the uncertainty on the angular response was conservatively set between -5^∘^ and 5^∘^ to account for potential small misalignments of the detector system. In operational conditions, however, a larger uncertainty, up to 60^∘^ or more, must be considered.

The uncertainty on the accuracy of the clustering algorithm has been evaluated based on previous work, up to 120 keV ^3^. The standard deviation of the observed shift in the number of counts was calculated over 10 keV bins and further used to determine a type A statistical uncertainty, associated on the number of count variation due to the clustering algorithm.

Conversion coefficients from ICRU Report 95 or ICRP Publication 74 are deemed to have no uncertainties.

The uncertainty associated with analog pileup, $u(f_{analog})$, was estimated using Equation 2, where $\dot{\Phi}$ is fluence rate incident on the Timepix4 detector, $n_{pixel}=229,376$ corresponds to the total number of pixels in the detector matrix, and $ToT_{max}$ is the *ToT* value corresponding to the highest detected energy during measurement. In this study, $ToT_{max}=3429 \mathrm{ns}$, corresponding to a photon energy deposition of 120 keV in a single pixel. The probability that no analog pileup occurs in the detector can be approximated using a paralysable model, commonly employed to describe the dead time of radiation detectors, in which a new hit cannot overlap with a previous one ^4^. Equation 2 is therefore expressed as the reversed probability associated with a paralysable model.

$u\left( f_{analog} \right)=1-e^{-\frac{\dot{\Phi}}{n_{pixels}}ToT_{max}}$ (2)

As described in Section 1.4 from Material and methods, the uncertainty due to digital pileup, $u(f_{digital})$, was estimated as the digital pileup rate for each measurement. This rate was computed directly from the pileup bit contained in each data set recorded by the Timepix4 detector.

According to the JCGM GUM-6:2020 guidelines ^5^, and given that none of the variables described in Equation 1 are correlated, the combined uncertainty for each component of the sum can be computed using a first-order Taylor series expansion, as expressed in Equation 3, where X represents the variables defined in Table 1. The total uncertainty is therefore computed as the quadratic sum of each bin uncertainty, since each bin contributes independent noise to the total quantity ^5^.

$\frac{u(K_{a}\left( E \right))}{K_{a}(E)}=\sqrt{\sum_{X} \left( \frac{u\left( X\left( E \right) \right)}{X\left( E \right)} \right)^{2}}$ (3)

**Supplementary Tables**

**Table 1 – Contributions of the variables involved in dose calculations with the Timepix4 detector to the global uncertainty on the measurement.**

| Variable | Description | Relative value (%) | |
| --- | --- | --- | --- |
|  |  | Type A | Type B |
| $\boldsymbol{u(ToT)}$ | Statistical uncertainty on *ToT* measurement, averaged over the whole pixel matrix, estimated using *n*=20 repeated measurements. | < 1.23 * | - |
| $\boldsymbol{u(}\boldsymbol{\epsilon}_{\boldsymbol{Si}}\boldsymbol{)}$ | Uncertainty on the first ionisation energy of silicon, from Pehl et al ^6^. | - | 0.55 |
| $\boldsymbol{u(}\boldsymbol{f}_{\boldsymbol{fit}}\boldsymbol{)}$ | Uncertainty on the calibration fit, determined using a reduced chi-squared test and averaged over the whole matrix. | < 0.05 | - |
| $\boldsymbol{u(}\boldsymbol{f}_{\boldsymbol{temp}}\boldsymbol{)}$ | Uncertainty due to ambient temperature variation, estimated from the response shift observed between 18^∘^C and 22^∘^C, reported in previous work ^7^. | - | 0.07 |
| $\boldsymbol{u(N)}$ | Statistical uncertainty on the detector counting capability, averaged over the whole matrix and estimated with *n*=20. | < 2.64 * | - |
| $\boldsymbol{u(}\boldsymbol{\mu}_{\boldsymbol{ph}}\boldsymbol{)}$ | Uncertainty from Monte-Carlo simulations of detection efficiency, represented as the statistical uncertainty on the number of photons contributing to the simulated efficiency values. | < 10^-4^ * | - |
| $\boldsymbol{u(}\boldsymbol{f}_{\boldsymbol{ang}}\boldsymbol{)}$ | Uncertainty due to angular variation, estimated from previous work ^7^. | - | 1.75 |
| $\boldsymbol{u(}\boldsymbol{f}_{\boldsymbol{analog}}\boldsymbol{)}$ | Uncertainty induced due analog pileup, estimated using equation 2. | - | < 0.01 ** |
| $\boldsymbol{u(}\boldsymbol{f}_{\boldsymbol{digital}}\boldsymbol{)}$ | Uncertainty due to digital pileup, estimated by retrieving information contained in the pileup bit. | - | < 0.12 ** |
| $\boldsymbol{u(}\boldsymbol{f}_{\boldsymbol{clusters}}\boldsymbol{)}$ | Uncertainty due by the cluster reconstruction algorithm, evaluated per energy bin in a previous study ^3^. | < 1.54 * | - |

***** The maximum uncertainty value is displayed, as the uncertainty depends on the energy and is therefore different for each energy bin.

** The maximum uncertainty value is displayed, as the uncertainty on both pileups depends on the fluence rate to which the detector is exposed.

**Table 2 – Relative response *r* of the Timepix4 in terms of total air kerma** $\boldsymbol{K}_{\boldsymbol{a}}$**.**

|  | RQR3 | RQR5 | RQR7 | RQR9 | RQA3 | RQA5 | RQA7 | RQA9 |
| --- | --- | --- | --- | --- | --- | --- | --- | --- |
| r | **1.045** | **1.055** | **1.139** | **1.213** | **1.044** | **1.068** | **1.232** | **1.391** |

**Table 3 – Relative response *r* of the Timepix4 in terms of ambient dose** $\boldsymbol{H}^{\boldsymbol{*}}$**.**

|  | RQR3 | RQR5 | RQR7 | RQR9 | RQA3 | RQA5 | RQA7 | RQA9 |
| --- | --- | --- | --- | --- | --- | --- | --- | --- |
| r | **0.979** | **0.958** | **1.116** | **1.151** | **0.951** | **1.007** | **1.132** | **1.285** |

**Table 4 – Relative response *r* of the Timepix4 in terms of ambient dose equivalent** $\boldsymbol{H}^{\boldsymbol{*}}\boldsymbol{(10)}$**.**

|  | RQR3 | RQR5 | RQR7 | RQR9 | RQA3 | RQA5 | RQA7 | RQA9 |
| --- | --- | --- | --- | --- | --- | --- | --- | --- |
| r | **1.041** | **1.046** | **1.205** | **1.266** | **1.005** | **1.049** | **1.178** | **1.316** |

**Table 5 – Relative response *r* of the Timepix4 in terms of directional absorbed dose in local skin** $\boldsymbol{D}_{\boldsymbol{local skin}}^{\boldsymbol{'}}$**.**

|  | RQR3 | RQR5 | RQR7 | RQR9 | RQA3 | RQA5 | RQA7 | RQA9 |
| --- | --- | --- | --- | --- | --- | --- | --- | --- |
| r | **0.993** | **0.962** | **1.021** | **1.050** | **0.963** | **0.955** | **1.001** | **1.126** |

**Table 6 – Relative response *r* of the Timepix4 in terms of directional dose equivalent at 0.07 mm depth** $\boldsymbol{H'(}\boldsymbol{0.07,0}^{\boldsymbol{\circ}}\boldsymbol{)}$**.**

|  | RQR3 | RQR5 | RQR7 | RQR9 | RQA3 | RQA5 | RQA7 | RQA9 |
| --- | --- | --- | --- | --- | --- | --- | --- | --- |
| r | **1.062** | **1.083** | **1.201** | **1.277** | **1.058** | **1.086** | **1.256** | **1.407** |

**Table 7 – Relative response *r* of the Timepix4 in terms of directional absorbed dose in the lens of the eye**  $\boldsymbol{D}_{\boldsymbol{lens}}^{\boldsymbol{'}}$**.**

|  | RQR3 | RQR5 | RQR7 | RQR9 | RQA3 | RQA5 | RQA7 | RQA9 |
| --- | --- | --- | --- | --- | --- | --- | --- | --- |
| r | **0.990** | **0.988** | **1.098** | **1.157** | **1.009** | **1.032** | **1.172** | **1.325** |

**Table 8 – Relative response *r* of the Timepix4 in terms of directional equivalent at 3 mm depth** $\boldsymbol{H'(3,}\boldsymbol{0}^{\boldsymbol{\circ}}\boldsymbol{)}$**.**

|  | RQR3 | RQR5 | RQR7 | RQR9 | RQA3 | RQA5 | RQA7 | RQA9 |
| --- | --- | --- | --- | --- | --- | --- | --- | --- |
| r | **1.098** | **1.045** | **1.168** | **1.237** | **1.008** | **1.035** | **1.178** | **1.331** |

**Table 9 – Root mean square and maximum values of absolute residuals between the *K_a_* distribution published by Ankerhold and the *K_a_* distribution computed using the present paper method and the** $\boldsymbol{\Phi}_{\boldsymbol{E}}$ **distribution published by Ankerhold.**

|  | RMS residuals (%) | Higher residual (%) |
| --- | --- | --- |
| RQR3 | 0.39 | 1.17 |
| RQR5 | 0.40 | 1.41 |
| RQR7 | 0.38 | 1.48 |
| RQR9 | 0.41 | 1.39 |

**Table 10 – Root mean square and maximum values of absolute residuals between the measured spectra at 1 and 2.5 m for RQA beam qualities**

|  | RMS residuals (%) | Higher residual (%) |
| --- | --- | --- |
| RQA3 | 0.48 | 1.56 |
| RQA5 | 0.46 | 1.51 |
| RQA7 | 0.22 | 0.60 |
| RQA9 | 0.57 | 1.76 |

**Supplementary discussion**

**Accuracy of the dose computation method**

Table 9 highlight a strong agreement between the reference and the computed distributions in terms of *K_a_.* The RMS, consistently below 0.5 % indicates only minor to no variations, most likely coming from slightly different values in terms of photoelectric and Compton effect cross-sections. The higher residual value is consistently located at 35 keV across all beam qualities tested. In the same way, this recurrence most probably highlights minor discrepancies in cross-sections or interpolation results. The method described in this manuscript is as accurate as the one described by Ankerhold ^1^ to reconstruct dose quantities from energy spectra.

**Homogeneity of the X-ray beam**

Table 10 shows an agreement within 0.5% between RQA spectra acquired at 1 m and 2.5 m, and maximum residuals below 1.76 %, occurring between 59 and 67 keV and corresponding to the K-lines produced by the tungsten anode. The combination of low RMS values and maximum residuals well below the per-bin indicates that the spectra at 1 and 2.5 m are sufficiently consistent. This agreement supports the use of the same conversion coefficients for both the RQR and the RQA series at 2.5 m.

**References**

1. Ankerhold, U. Catalogue of X-ray spectra and their characteristic data : ISO and DIN radiation qualities, therapy and diagnostic radiation qualities, unfiltered X-ray spectra. *PTB-OAR* https://doi.org/10.7795/110.20190315B (2000) doi:10.7795/110.20190315B.

2. International Electrotechnical Commission. Medical diagnostic X-ray equipment - Radiation conditions for use in the determination of characteristics. (2005).

3. Genetay, T. *et al.* RQR beam qualities measurement with the Timepix4 detector. *Radiat. Meas.* **191**, 107570 (2026).

4. Knoll, G. F. *Radiation Detection and Measurement*. (John Wiley & sons, Hoboken, NJ, USA, 2008).

5. Joint Committee for Guides in Metrology. *Guide to the Expression of Uncertainty in Measurement — Part 6: Developing and Using Measurement Models*. (2020).

6. Pehl, R. H., Goulding, F. S., Landis, D. A. & Lenzlinger, M. Accurate determination of the ionization energy in semiconductor detectors. *Nucl. Instrum. Methods* **59**, 45–55 (1968).

7. Genetay, T. *et al.* Characterisation of the Timepix4 silicon sensor detector for low energy X-ray applications. *Nucl. Instrum. Methods Phys. Res. Sect. Accel. Spectrometers Detect. Assoc. Equip.* **1083**, 171163 (2026).
